# Age- and BMI-Associated Expression of Angiogenic Factors in White Adipose Tissue of Children

**DOI:** 10.3390/ijms20205204

**Published:** 2019-10-21

**Authors:** Niklas Gaebler, Benedikt Haggenmüller, Melanie Kapapa, Alexandre Serra, Daniel Tews, Jan-Bernd Funcke, Stephanie Brandt, Valentin Ioannidis, Michael Schön, Peter Möller, Klaus-Michael Debatin, Martin Wabitsch, Pamela Fischer-Posovszky

**Affiliations:** 1 Division of Pediatric Endocrinology and Diabetes, Department of Pediatrics and Adolescent Medicine, University Medical Center Ulm, 89075 Ulm, Germany; niklas.gaebler@uni-ulm.de (N.G.); benedikt.haggenmueller@uniklinik-ulm.de (B.H.); daniel.tews@uniklinik-ulm.de (D.T.); jan-bernd.funcke@UTsouthwestern.edu (J.-B.F.); stephanie.brandt@uniklinik-ulm.de (S.B.); martin.wabitsch@uniklinik-ulm.de (M.W.); 2 Division of Pediatric Surgery, Department of Surgery, University Medical Center Ulm, 89075 Ulm, Germany; melanie.kapapa@uniklinik-ulm.de (M.K.); alexandre.serra@uniklinik-ulm.de (A.S.); 3 Institute of Anatomy and Cell Biology, Ulm University, 89081 Ulm, Germany; valentin.ioannidis@uni-ulm.de (V.I.); michael.schoen@uni-ulm.de (M.S.); 4 Institute of Pathology, University Medical Center Ulm, 89081 Ulm, Germany; peter.moeller@uniklinik-ulm.de; 5 Department of Pediatrics and Adolescent Medicine, University Medical Center Ulm, 89075 Ulm, Germany; klaus-michael.debatin@uniklinik-ulm.de

**Keywords:** adipose tissue, angiogenesis, adipogenesis, obesity, remodeling, vasculature

## Abstract

The growth of adipose tissue and its vasculature are tightly associated. Angiogenic factors have been linked to obesity, yet little is known about their expression during early childhood. To identify associations of angiogenic factors with characteristics on individual and tissue level, subcutaneous white adipose tissue samples were taken from 45 children aged 0–9 years undergoing elective surgery. We measured the expression of vascular endothelial growth factor A (VEFGA), fibroblast growth factor 1 and 2 (FGF1, FGF2), angiopoietin 1 and 2 (ANGPT1, ANGPT2), TEK receptor tyrosine kinase (TEK), and von Willebrand factor (VWF). In addition, we determined the mean adipocyte size in histologic tissue sections. We found positive correlations of age with FGF1 and FGF2 and a negative correlation with ANGPT2, with pronounced differences in the first two years of life. FGF1, FGF2, and ANGPT1 correlated positively with adipocyte size. Furthermore, we identified a correlation of ANGPT1 and TEK with body mass index-standard deviation score (BMI-SDS), a measure to define childhood obesity. Except for ANGPT2, all angiogenic factors correlated positively with the endothelial marker VWF. In sum, our findings suggest that differences related to BMI-SDS begin early in childhood, and the analyzed angiogenic factors possess distinct roles in adipose tissue biology.

## 1. Introduction

The prevalence of childhood obesity is increasing alarmingly all over the world, making it one of the most serious public health challenges of the 21st century [1]. Obesity in children often persists into adulthood [2] and increases the risk of developing cardiovascular and metabolic disorders [3].

Obesity is characterized by the accumulation of white adipose tissue (WAT). In general, the expansion of WAT can occur through an increase in adipocyte number (hyperplasia) or an increase in adipocyte size (hypertrophy) [4]. In humans, WAT develops already in utero. It becomes discernable between week 14 and 16 after gestation, and after 28 weeks, fat lobuli can be detected at all typical designated WAT locations [5,6]. At birth, both subcutaneous and visceral WAT depots are already well developed and account for approximately 16% of total body weight [7]. Postnatally, the fastest growth of WAT can be observed in the first year of life at the age of four to six months [8,9]. At the age of 18 months, WAT mass accounts for approximately 28% of body weight [10]. 

The development and growth of WAT is tightly associated with angiogenesis. Already in 1980, Hausman et al. described that the formation of primary fat lobuli during prenatal WAT development goes along with the sprouting of blood vessels [11]. A small network of capillaries seemed to serve as a key structure for the adipogenic differentiation of tissue resident progenitor cells and thereby defined the emerging fat lobuli [6,12]. Decades later, progenitor cells were indeed identified in the vasculature of WAT, establishing the vascular wall as a local progenitor cell niche [13]. Nowadays, it is well accepted that adipogenesis and angiogenesis are spatiotemporally associated and interdependent on a molecular level [14]. Actually, antiangiogenic strategies are discussed as a novel treatment option for obesity [14].

Intriguingly, obesity already develops early in life, commencing from the age of two years [15]. A study by Landgraf et al. demonstrated that obese children had larger adipocytes and a higher number of adipocytes at the age of six years [16]. However, the knowledge on adipose tissue cellularity at earlier ages is scarce.

Given the fact that WAT mass alters rapidly and enormously during the first 18 months of life, which is shortly before the initial onset of obesity was observed [15,16], we aimed to study WAT cellularity in early childhood with a specific focus on adipocyte size. As adipogenesis and angiogenesis are closely intertwined, we further sought to determine the expression of angiogenic factors to elucidate possible associations to hallmarks of WAT growth as well as relationships to age and body weight status.

## 2. Results

### 2.1. General Characteristics of the Ulm Childhood Adipose Tissue Collective

An overview of the general characteristics of the Ulm Childhood Adipose Tissue Collective is presented in Table 1. Tissue samples were collected during elective surgery, mostly hernia repairs, which are mainly performed in children under the age of one year, but also orchidopexies among few others (further details in Materials and Methods). The collective comprises 36 boys and 9 girls. As cryptorchidisms are exclusive to and inguinal hernias more common in boys [17], the uneven sex distribution was unsurprising and is comparable to similar cross-sectional studies [16,18]. The age in the collective ranged from −0.04 to 9.23 years. Four patients had a slightly negative age because of the age correction due to preterm delivery. The marked difference between the mean and median age is due to an unequal age distribution in the collective. Of the patients, 27 of 45 were younger than one year, making infants a considerably large group within the collective. Both inguinal hernias and cryptorchidisms are linked to prematurity [17,19], explaining the high number of children born preterm and infants. 

Height, weight, and BMI correlated positively with age, and their distributions in the collective were reminiscent of published percentile curves when plotted against age (data not shown). Applying the definitions of underweight (3rd–10th BMI-percentile), severe underweight (<3rd BMI-percentile), overweight (90th–97th BMI-percentile), and obesity as >97th BMI-percentile) by the reference population [20], five children were underweight, three overweight, and one obese. 

For all further analyses, the age and sex-adjusted BMI data are given as BMI-SDS (standard deviation scores) in which zero resembles the median BMI in the reference population, and numbers higher or lower describe how many standard deviations the individual BMI is above or below this median.

### 2.2. Tissue Cellularity

**Figure 1:**
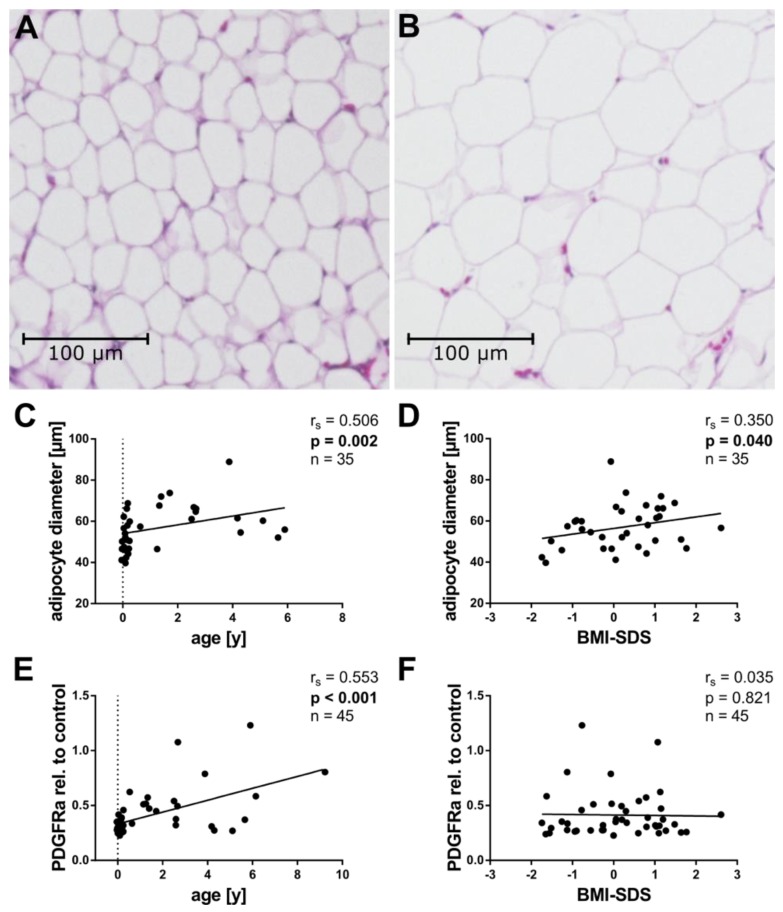
Association of adipocyte size and PDGFRa expression with age and BMI-SDS. (**A**,**B**) Pictures of representative adipose tissue sections stained with haematoxylin and eosin from a patient with small (**A**) and a patient with large (**B**) adipocytes. (**C**,**D**) Adipocyte diameter was plotted against age (**C**) and BMI-SDS (**D**). Adipocyte size correlated positively with age and BMI-SDS in our collective (**C**,**D**). (**E**,**F**) Total RNA was isolated, reverse transcribed, and subjected to qPCR analysis using specific primer pairs as indicated. PDGFRa gene expression was calculated as described in Methods and plotted against age. PDGFRa expression correlated positively with age (**E**) but not with BMI-SDS (**F**) in the collective. Spearman correlation coefficient *r_s_* and *p* value, as well as number of subjects, are given in each scatter plot. Significant *p* values (*p* < 0.05) are indicated in bold. Trend lines were calculated as linear regressions.

Adipocyte size is increased in obese WAT and linked to adverse metabolic functions [4]. To investigate adipocyte size, we analyzed the mean adipocyte Feret diameter in H&E-stained tissue sections. In total, the tissue samples of 35 patients were large enough to allow such a histologic assessment. Figure 1 shows two representative tissue sections, one from a patient with small (Figure 1A) and one with large adipocytes (Figure 1B).

Adipocyte size is known to increase with age [21], and indeed, adipocyte size correlated positively with age in our collective (Figure 1C). Moreover, adipocyte size correlated positively with BMI-SDS, linking it to the weight status (Figure 1D).

In order to gain more insight into the cellularity of the tissue, we measured the mRNA expression of PDGFRa, a well described marker of adipocyte precursor cells [22]. Interestingly, we detected a positive correlation of PDGFRa with age (Figure 1E), but not with BMI-SDS (Figure 1F).

### 2.3. Angiogenic Factors and Age

**Figure 2:**
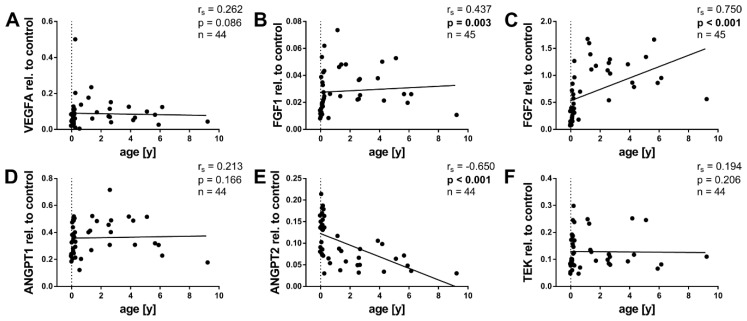
Association of angiogenic gene expression in pediatric WAT with age. Total RNA was isolated, reverse transcribed, and subjected to qPCR analysis using specific primer pairs as indicated. Respective gene expression was calculated as described in Methods and plotted against age. (**A**) VEGFA expression showed no significant correlation with age. (**B**,**C**) FGF1 and FGF2 expression, however, correlated positively with age. (**D**–**F**) While ANGPT1 and the receptor TEK showed no significant correlation with age (**D**,**F**), ANGPT2 displayed a negative correlation (**E**). Spearman correlation coefficient *r_s_* and *p* value, as well as number of subjects, are given in each scatter plot. Significant *p* values (*p* < 0.05) are indicated in bold. Trend lines were calculated as linear regressions.

**Figure A2:**
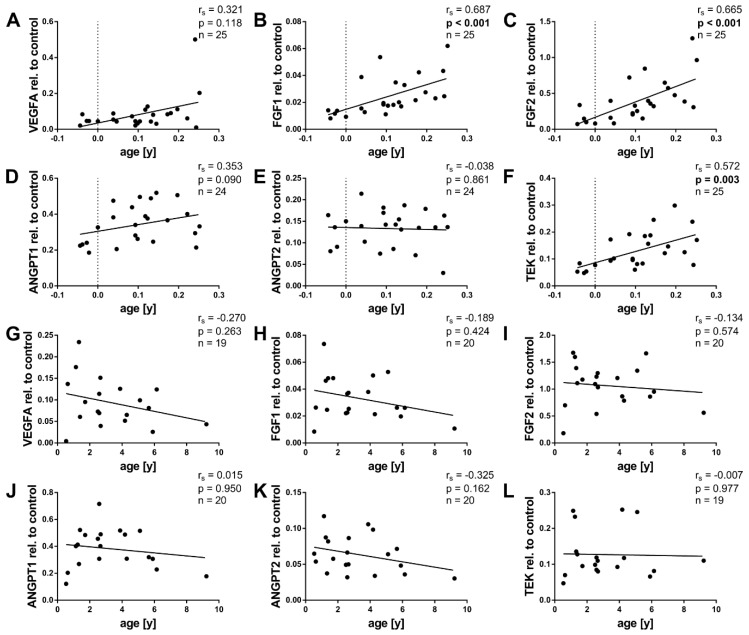
Association of angiogenic gene expression with age in the WAT of infants <6 and children ≥6 months. Total RNA was isolated, reverse transcribed, and subjected to qPCR analysis using specific primer pairs as indicated. Respective gene expression was calculated as described in Methods and plotted against age. (**A**) As in the correlation analysis with all children, VEGFA expression showed no significant correlation with age in the infants <6 months. (**B**,**C**) Both FGF1 and FGF2 expression still correlated positively with age when performing the analyses with the infants younger than 6 months only. (**D**) ANGPT1 showed no significant correlation with age. (**E**,**F**) In contrast to the analysis with all children, ANGPT2 showed no significant correlation with age (**E**), while TEK correlated positively with age in the infants <6 months. (**F**). (**G**–**L**) There were no significant correlations of the analyzed angiogenic factors with age in the group of children ≥6 months. Spearman correlation coefficient *r_s_* and *p* value, as well as number of subjects, are given in each scatter plot. Significant *p* values (*p* < 0.05) are indicated in bold. Trend lines were calculated as linear regressions.

As adipogenesis and angiogenesis are intertwined processes, we investigated the mRNA expression of angiogenic factors. Since WAT growth changes throughout childhood [23], we first analyzed their association with age. The supposedly best-known angiogenic factor, VEGFA, did not correlate significantly with age (Figure 2A). FGF1 and FGF2, in turn, displayed positive correlations with age (Figure 2B,C), with a strong correlation coefficient of r_s_ = 0.750 for FGF2. ANGPT1 and its receptor TEK did not correlate with age (Figure 2D,F). ANGPT2, in contrast, correlated negatively with age (Figure 2E). Interestingly, the factors with the strongest correlations, FGF2 and ANGPT2, showed the greatest differences between infants <6 months and children ≥6 months. Figure A2 in the Appendix A presents the angiogenic expression of the infants <6 months in more detail.

### 2.4. Angiogenic Factors and BMI-SDS

**Figure 3:**
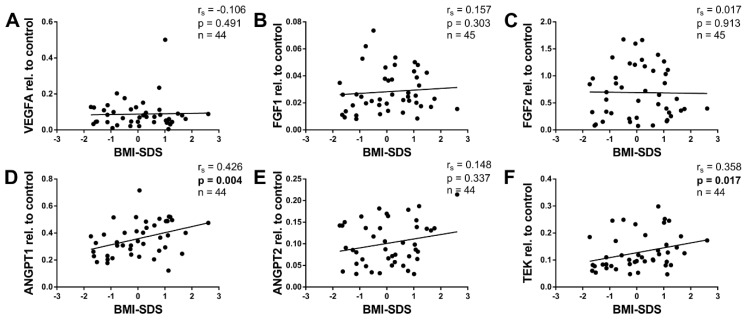
Association of angiogenic gene expression in pediatric WAT with BMI-SDS. Total RNA was isolated, reverse transcribed, and subjected to qPCR analysis using specific primer pairs as indicated. Respective gene expression was calculated as described in Methods and plotted against BMI-SDS. (**D**,**F**) ANGPT1 and the receptor TEK correlated positively with BMI-SDS. (**A**–**C**,**E**) In contrast, VEGFA, FGF1, FGF2, and ANGPT2 showed no significant correlation. Spearman correlation coefficient *r_s_* and *p* value, as well as number of subjects, are given in each scatter plot. Significant *p* values (*p* < 0.05) are indicated in bold. Trend lines were calculated as linear regressions.

WAT angiogenesis is altered in obese human adults as well as in mice [24,25]. To our knowledge, however, there are no reports addressing this question in children. In the present study, only a few children were overweight, and one child was obese. It was, therefore, not possible to determine the angiogenic expression in obesity per se. However, the BMI-SDS can be considered a measurement of the weight status. We, therefore, investigated associations between the mRNA expression of angiogenic factors and BMI-SDS in our collective. Figure 3 presents the angiogenic factors plotted against BMI-SDS. 

As with age, VEGFA displayed no correlation with BMI-SDS (Figure 3A). Furthermore, FGF1 and FGF2 did not correlate with BMI-SDS (Figure 3B,C). In contrast, ANGPT1 and TEK correlated positively with BMI-SDS (Figure 3D,F), while ANGPT2 showed no correlation (Figure 3E). Intriguingly, factors that correlated with age (FGF1, FGF2, and ANGPT2) did not correlate with BMI-SDS, while those that correlated positively with BMI-SDS (ANGPT1 and TEK) showed no association with age. This suggests that some angiogenic factors might be regulated during physiologic growth over time, while others could be involved in excessive weight gain.

### 2.5. Angiogenic Factors and Adipocyte Size

**Figure 4:**
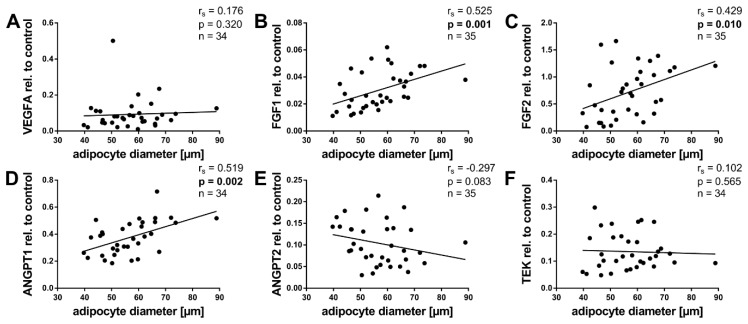
Association of angiogenic gene expression in pediatric WAT with adipocyte size. Total RNA was isolated, reverse transcribed, and subjected to qPCR analysis using specific primer pairs as indicated. Respective gene expression was calculated as described in Methods and plotted against adipocyte diameter. (**B**–**D**) FGF1, FGF2, and ANGPT1 showed a positive correlation with adipocyte diameter. (**A**,**E**,**F**) VEGFA, ANGPT2, and TEK, in contrast, showed no significant correlations with adipocyte diameter. Spearman correlation coefficient *r_s_* and *p* value, as well as number of subjects, are given in each scatter plot. Significant *p* values (*p* < 0.05) are indicated in bold. Trend lines were calculated as linear regressions.

We furthermore performed correlation analyses to elucidate associations between adipocyte size and angiogenic gene expression. The results are presented in Figure 4. As with age and BMI-SDS, VEGFA showed no significant correlation with adipocyte size (Figure 4A). However, FGF1, FGF2, and ANGPT1 correlated positively with adipocyte size (Figure 4B–D). ANGPT2 and TEK in contrast did not correlate with adipocyte size (Figure 4E,F). However, ANGPT2 showed a negative trend.

### 2.6. Angiogenic Factors and von Willebrand Factor

To assess tissue vascularization, we determined the mRNA expression of von Willebrand factor (VWF), which is specifically expressed and constitutively secreted by endothelial cells [26,27]. VWF expression did not correlate with age (not shown); however, it correlated positively with BMI-SDS (r_s_ = 0.343, *p* = 0.021, *n* = 45, not shown), thus suggesting an association between vascularity and the weight status.

**Figure 5:**
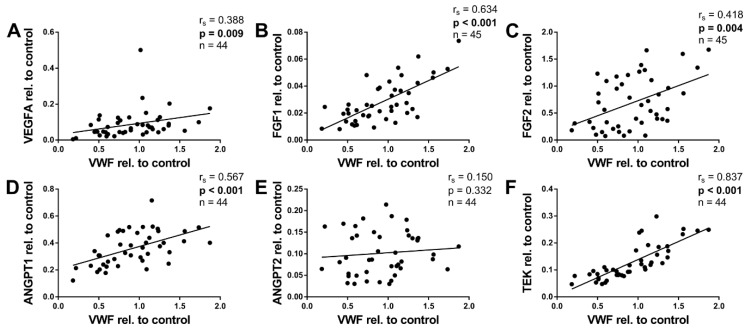
Association of angiogenic gene expression in pediatric WAT with VWF expression. Total RNA was isolated, reverse transcribed, and subjected to qPCR analysis using specific primer pairs as indicated. Respective gene expression was calculated as described in Methods. (**A**–**D**) VEGFA, FGF1, FGF2, and ANGPT1 correlated positively with VWF. (**E**) There was no significant correlation between ANGPT2 and VWF. (**F**) TEK, in turn, showed the strongest correlation with VWF. Spearman correlation coefficient *r_s_* and *p* value, as well as number of subjects, are given in each scatter plot. Significant *p* values (*p* < 0.05) are indicated in bold. Trend lines were calculated as linear regressions.

VEFGA, FGF1, FGF2, and ANGPT1 all correlated positively with VWF expression (Figure 5A–D). Moreover, TEK showed a very strong correlation coefficient of r_s_ = 0.837, which is not surprising considering that TEK is similarly almost exclusively expressed by endothelial cells [28]. Only ANGPT2 did not correlate with VWF (Figure 5E). 

### 2.7. Angiogenic Factors in Infants <6 Months

**Figure A3:**
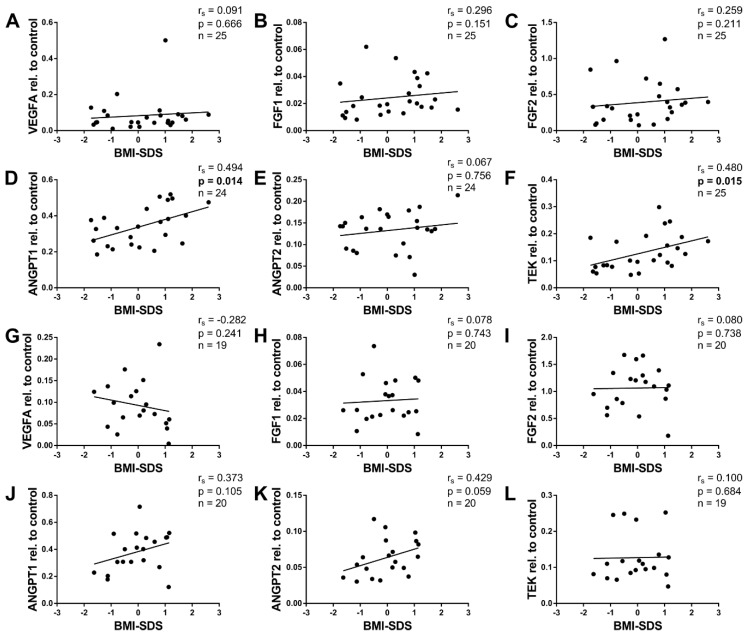
Association of angiogenic gene expression with BMI-SDS in the WAT of infants <6 and children ≥6 months. Total RNA was isolated, reverse transcribed, and subjected to qPCR analysis using specific primer pairs as indicated. Respective gene expression was calculated as described in Methods and plotted against BMI-SDS. (**A**–**F**) Like in the analysis with all children, ANGPT1 and the receptor TEK correlated positively with BMI-SDS in the infants <6 months (**D**,**F**), while VEGFA, FGF1, FGF2, and ANGPT2 showed no significant correlation (**A**–**C**,**E**). (**G**–**L**) We found no significant correlation of angiogenic factors with BMI-SDS in the children ≥6 months. Spearman correlation coefficient *r_s_* and *p* value, as well as number of subjects, are given in each scatter plot. Significant *p* values (*p* < 0.05) are indicated in bold. Trend lines were calculated as linear regressions.

**Figure A4:**
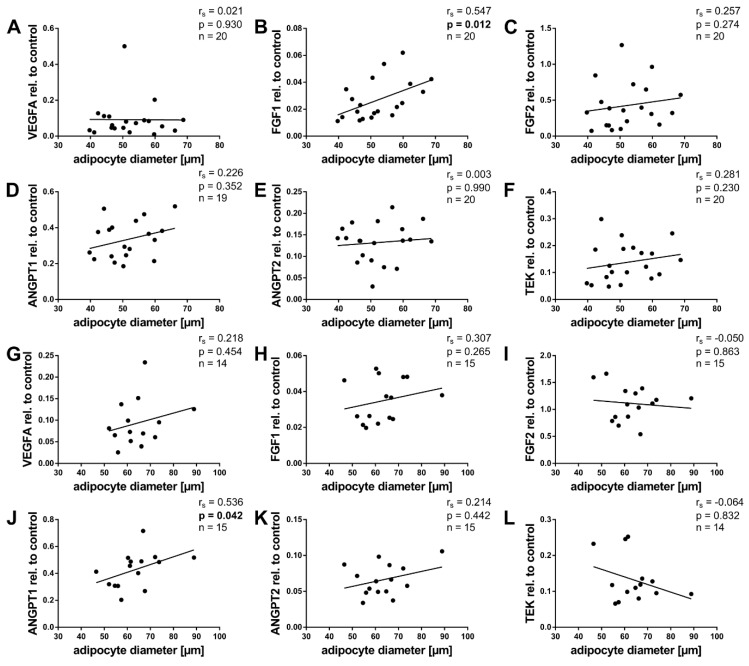
Association of angiogenic gene expression with adipocyte size in the WAT of infants <6 and children ≥6 months. Total RNA was isolated, reverse transcribed, and subjected to qPCR analysis using specific primer pairs as indicated. Respective gene expression was calculated as described in Methods and plotted against adipocyte diameter. (**B**) Only FGF1 correlated positively with adipocyte size in the infants <6 months. (**C**,**D**) Both FGF2 and ANGPT1, which correlated positively with adipocyte diameter in the analysis with all children, did not correlate significantly with adipocyte size in the infants <6 months. (**A**,**E**,**F**) VEGFA, ANGPT2, and TEK showed no significant correlations with adipocyte diameter in the <6 months group. (**G**–**L**) In contrast, in the children ≥6 months, only ANGPT1 correlated positively with adipocyte diameter (**J**), while there was no correlation of the other angiogenic factors (**G**–**I**,**K**,**L**). Spearman correlation coefficient *r_s_* and *p* value, as well as number of subjects, are given in each scatter plot. Significant *p* values (*p* < 0.05) are indicated in bold. Trend lines were calculated as linear regressions.

**Figure A5:**
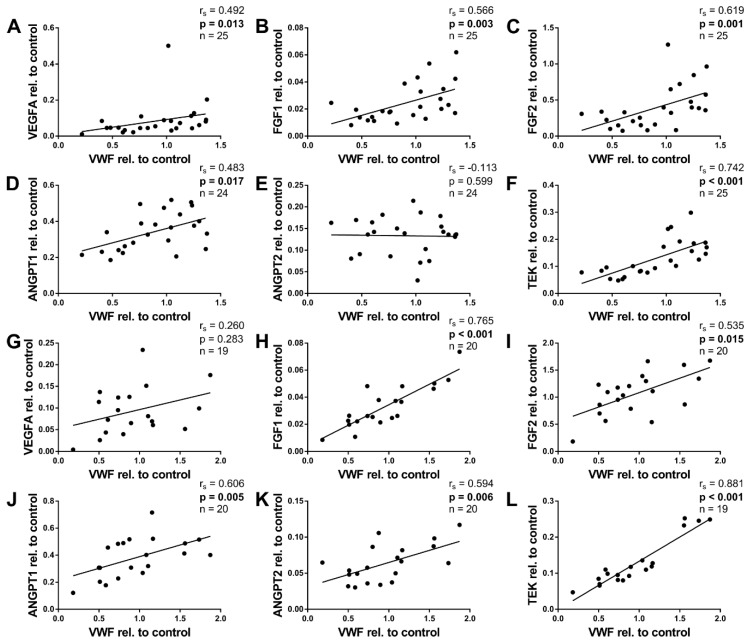
Association of VWF with angiogenic gene expression in the WAT of infants <6 and children ≥6 months. Total RNA was isolated, reverse transcribed, and subjected to qPCR analysis using specific primer pairs as indicated. Respective gene expression was calculated as described in Methods. (**A**–**F**) Like in the analysis with all children, all analyzed angiogenic factors, with the exception of ANGT2, correlated positively with VWF when performing the analysis with the infants <6 months only. (**G**–**L**) In the children ≥6 months VEGFA did not correlate significantly with VWF (**G**). In contrast to the analyses with the infants <6 months and all children, ANGPT2 correlated positively with VWF in the ≥6 months group (**K**). (**H**–**J**,**L**) As in the infants <6 months FGF1, FGF2, ANGPT1, and L also correlated positively with VWF in the children ≥6 months. Spearman correlation coefficient *r_s_* and *p* value, as well as number of subjects, are given in each scatter plot. Significant *p* values (*p* < 0.05) are indicated in bold. Trend lines were calculated as linear regressions.

Most of the children in the present study were infants younger than six months (*n* = 25). As the first months of postnatal development are a pivotal phase in WAT development, and we were intrigued to see if the angiogenic factors also showed significant correlations when examining these infants only. Thus, we performed all analyses anew with only infants <6 months and for comparison, with children ≥6 months. The results are presented in Table 2, and the respective Figure A2, Figure A3, Figure A4 and Figure A5 can be found in the Appendix A.

In sum, most results in the infants <6 months were very similar to those with all children, suggesting that most of the observed associations already exist in the first few months of life. This links the angiogenic expression to a crucial phase in WAT development and further emphasizes the tight association of the WAT and its vasculature. 

However, there were a few differences in the subgroup analyses, which are worth mentioning. The negative correlation of ANGPT2 with age, found with all children, was no longer observed in the <6 months group. In contrast, TEK correlated positively with age in the infants <6 months, while there was no correlation with TEK in the analysis with all children or in the group ≥6 months. As TEK is almost exclusively expressed by endothelial cells and VWF also correlates positively with age in the infants <6 months, the positive correlation might stem from a growth in endothelial cell numbers rather than an upregulation in endothelial cells themselves. The difference in ANGPT2 expression between the analysis of the infants <6 months and that of all children could be due to a distinct regulation or role of the factor. Interestingly, in the group ≥6 months, ANGPT2 correlated positively with VWF.

## 3. Discussion

In this study, we analyzed the mRNA expression of angiogenic factors in the white adipose tissue samples of children undergoing elective surgery. To identify associations of the angiogenic factors, we performed correlation analyses with anthropometric parameters and the adipocyte size. The median age of the children was 0.24 years. In fact, almost two thirds, the children were in their first year of life. To our knowledge, this is the first study addressing adipose tissue cellularity and angiogenesis in children at such a young age.

The growth and expansion of adipose tissue occurs by either an increase in adipocyte numbers or adipocyte volume. Back in the 1970s, Knittle et al. provided evidence that the total number of adipocytes increases tremendously until early adulthood [29]. Importantly, obese children have more adipocytes already two years after birth [29]. This difference seems to be mediated by an enhanced proliferation of stromal-vascular cells in adipose tissue of obese children, whereas their adipogenic differentiation capacity is not altered [16]. Due to the very young age of our subjects, the absolute adipose tissue sample size was very limited in our study. We managed to isolate stromal-vascular cells from a small subset of children but were not able to generate reliable data on ex vivo proliferation and adipogenic differentiation capacity or composition of the stromal-vascular fraction via flow cytometry. However, the material we retrieved was sufficient to isolate total RNA of 45 subjects and to perform histologic analyses of 35 patients.

The size of adipocytes is known to increase with age during childhood [16,21]. In line with this, adipocyte size correlated positively with age in our collective (Figure 1C). We also found a positive correlation of adipocyte size with the age and sex-adjusted BMI-SDS (Figure 1D). This latter finding reinforces the presumption that obesity-associated adipocyte hypertrophy begins in early childhood and is in line with a recent report in older children [16]. We want to point out that we determined adipocyte size by measuring the Feret diameter in the two-dimensional plane. The method will generally not resemble the true diameter of the adipocyte and, therefore, must be considered as an approximation. Hence, the values cannot be equalized with those determined by other methods of cell-size-determination, such as Coulter counting. Furthermore, we might oversee very small adipocytes present in children. The cell sizes in our collective are nonetheless comparable to those measured by Tam et al., who also determined the adipocyte diameter in histologic sections [18].

WAT is quite vascularized, with each adipocyte being connected to at least one capillary [25]. This is crucial to guarantee the supply of adipocytes with oxygen and nutrients and the proper exchange of adipose tissue signals with other organ systems of the body. Vascular structures are also a major prerequisite for WAT development. Crandall et al. described that adipocyte development in the fetus is temporally and spatially related to capillary growth and demonstrated that arteriolar development precedes the occurrence of adipocytes [30]. The growth of WAT involves the remodeling of the vascular network, including both dilation and remodeling of existing capillaries to support adipocyte hypertrophy, but also neovascularization in the case of adipocyte hyperplasia [25,31]. Adipocytes and endothelial cells are intimately connected, enabling the dynamic co-regulation of adipogenesis and angiogenesis by autocrine and paracrine signals. Both proangiogenic and antiangiogenic factors are produced and secreted by fat cells, and their balance is tightly regulated [25].

In our study, the expression of VWF served as a surrogate marker for tissue vascularity because it is expressed only in endothelial cells in WAT [26,27]. 

In our collective, VWF expression did not correlate with age. However, we found a positive correlation between VWF and BMI-SDS, suggesting an association between tissue vascularity and the weight status. This finding stresses the concept of concomitant growth of adipose tissue and its vasculature. In line with this, VWF is elevated in the plasma of obese children, and there is evidence for a differential expression in microarray studies [32,33,34,35]. 

The angiogenic process involves multiple factors. To identify angiogenic factors present in the adipose tissue of young children and to find associations with characteristics on individual and tissue level, we measured the mRNA expression of VEGF, FGF1, FGF2, and the angiopoietins ANGPT1 and ANGPT2, as well as their receptor TEK (also known as TIE-2). 

VEGFA is an endothelial-specific mitogen which acts in a paracrine manner and is essential for vascular development [36,37,38,39,40]. VEGFA is the most abundant member of the VEGF-family in human WAT [41].

In our collective, VEGFA expression correlated with none of the anthropometric parameters. This is in line with results published by Tam et al., who found no differences in the VEFG expression between obese and lean children [18]. While we found no changes in VEFGA expression with age or the weight status, this does not exclude a pivotal role of VEGFA in the WAT. In fact, VEGFA expression showed a positive correlation with VWF, linking it to endothelial cells. It, moreover, correlated positively with FGF1, FGF2, and TEK expression and negatively with ANGPT2 expression (data not shown), suggesting associations to the other angiogenic systems analyzed in the present study.

Both FGF1 and FGF2 correlated positively with age. The greatest differences in the expression seemed to occur in the first year of life (Figure 2B,C and Figure A2B,C). This falls together with an important phase in the development of the WAT. The total fat mass rises from 0.7 kg at birth to 2.8 kg at the age of one year, corresponding to an increase in body fat percentage from 16% to 28% [23]. Both FGFs correlated positively with adipocyte size (Figure 4B,C). For FGF2, this has previously been shown in cattle [42]. We found no associations with BMI-SDS.

FGF1 and FGF2 are endothelial mitogens that act in a paracrine manner [43,44,45,46]. In contrast to VEGFA, the FGFs are involved in a multitude of processes. Both FGF1 and FGF2 enhance adipogenesis [47,48,49,50]. Thus, the positive correlation with age and adipocyte size might be due to a pronounced adipogenesis. However, as FGF1 and FGF2 showed a positive correlation with VWF, we assumed at least a partial involvement in angiogenesis. Of all factors analyzed in the study, FGF2 was the factor with the lowest CT values in the qPCR analysis, demonstrating high expression levels. This furthermore implied it to play a central role in WAT development.

ANPGT2 should not be considered as a pro- or antiangiogenic factor per se. While gene-targeting studies proposed ANGPT2 as a TEK antagonist [51], others have reported an activation of TEK depending on concentration [52] and context [53], but to a lesser extent than ANGPT1 [54]. Whether ANGPT2 acts pro- or antiangiogenic also depends on the presence of other angiogenic factors, notably VEGFA [51,55,56]. ANGPT2 causes endothelial destabilization [57,58] and pericyte loss [59,60], which are thought to be essential steps in initiating angiogenic sprouting. 

ANGPT2 was the only factor that correlated negatively with age in our collective (Figure 2E). The greatest difference in its expression was found between infancy and early childhood. Thus, ANGPT2 expression contrasted the expression pattern of the FGFs. However, in the infants <6 months, we found no significant correlation (Figure A2E). We found no correlation of ANGPT2 with BMI-SDS. ANGPT2 was, moreover, the only angiogenic factor which did not correlate with VWF (Figure 5E). ANPGT2 and VWF are both expressed by endothelial cells and stored in the same subcellular organelles, the Weibel-Palate bodies [61]. This finding was thus rather unexpected. There is, however, evidence for a reciprocal regulation of ANGPT2 and VWF in endothelial cells [27], which provides a possible explanation for our observations. Interestingly, in the group ≥6 months, ANGPT2 correlated positively with VWF (Figure A5E). 

ANGPT1 expression did not correlate with age (Figure 2D). In contrast, it correlated positively with BMI-SDS, an age-independent measurement defining childhood obesity (Figure 3D). Furthermore, ANGPT1 also displayed positive correlations with adipocyte size and VWF (Figure 4D and Figure 5D). Taken together, ANGPT1 expression was associated with the weight status and vascularity alike. Moreover, its receptor TEK also correlated positively with BMI-SDS and VWF (Figure 3F and Figure 5F), reinforcing the idea that ANGPT1 signaling plays a role in WAT angiogenesis upon weight gain. It should be noted that the latter finding was expected, as TEK is considered to be mainly expressed by endothelial cells [28]. The likeness of the correlations of ANGPT1 and TEK may also stem from direct interactions, as ANGPT1 is able to upregulate TEK expression [62]. 

The association of ANGPT1 with BMI-SDS is in line with findings from Pasarica et al., who reported an elevated expression of ANGPT1 in the WAT of obese compared to lean patients [63]. In contrast, there are reports of a decreased expression in genetically-induced [64,65,66] and diet-induced [66] murine obesity models. Interestingly, Dallabrida et al. found that ANGPT1 expression correlated negatively with weight change, independent of direction [64]. Our study design did not provide data on the weight change of the patients. We were, thus, not able to address this question. 

Our study is unique as the majority of study participants were younger than six months at the time of surgery and tissue collection. There are, nonetheless, some limitations worth discussing. The design of our study is cross-sectional and, thus, restricts us to descriptive analyses. Due to the in part very small amounts of tissue, we chose qPCR to assess the expression of known angiogenic factors, limiting our results to the mRNA level, which may differ from the protein level. It should also be noted that we only analyzed the local tissue expression of the angiogenic factors, while circulating angiogenic factors are also expected to contribute to WAT angiogenesis [67].

Taken together, our study demonstrates that the adipose tissue is a dynamic organ in childhood with enormous remodeling in the first year of life. Our findings underline that differences in WAT related to the weight status begin early in childhood and that the angiogenic factors we analyzed presumably possess distinct roles in adipose tissue biology.

## 4. Materials and Methods

### 4.1. The Ulm Childhood Adipose Tissue Collective

Subcutaneous WAT samples were collected from 45 children undergoing elective surgery at the Division of Pediatric Surgery of the University Medical Center Ulm. The operations providing the samples were inguinal herniotomies (*n* = 34), orchidopexies (*n* = 6), umbilical herniotomies (*n* = 3), cholecystectomy (*n* = 1), and excision of an urachal cyst (*n* = 1). Systemic inflammation, malignant disease, genetic syndrome, and metabolic disorder were defined as exclusion criteria. The study was approved by the ethical review committee of the University of Ulm (ethics application No. 368/13, approval date 10 January 2014), and written informed consent was obtained from the patient’s parents in advance.

Data concerning the anthropometric parameters of the patients were taken from the record of the pediatric surgeons. For all patients whose gestational ages at birth were known, the corrected age was calculated by subtracting the difference between the due date (40 weeks) and gestational age at birth from the chronological age [68]. All ages used herein represent the corrected ages. To adjust the BMI data for age and sex, BMI-SDS (standard deviation scores) and percentiles were calculated using the original data and method from the population reference data published by Kromeyer-Hauschild et al. in 2001 [20].

### 4.2. Processing of the Adipose Tissue Samples

The adipose tissue samples were taken from the subcutaneous depot at the site of the incision at the beginning of the operation. Tissue that could not be macroscopically identified as adipose tissue, e.g., adjacent connective tissue, was removed under sterile conditions. One part of each tissue sample was then snap frozen for RNA isolation using liquid nitrogen, while the other was fixed for histological analysis using 4% formaldehyde. If the tissue sample was too small (<100 mg), only RNA isolation was performed.

### 4.3. Histological Sample Preparation

The tissue samples for the histological analysis were dehydrated using standard procedures, embedded in paraffin wax, and then sectioned into 3 µm thick slices. Haematoxylin and eosin (H&E) staining was performed using an Autostainer XL (Leica, Wetzlar, Germany).

### 4.4. Determination of the Adipocyte Size

Pictures of the microscopic slide were taken at 10× magnification using a BZ-9000 microscope (Keyence, Osaka, Japan). The adipocyte size was determined using Image J and the MRI Adipocyte macro in fields of 500 µm × 500 µm by manually circling intact adipocytes. The area and Feret diameter of every adipocyte were calculated. To rule out SVCs and adipocytes cut only in their very periphery, cells with areas smaller than 100 µm^2^ were excluded. Fields of 500 µm × 500 µm were analyzed until at least 100 valid adipocytes were measured for each patient. Then, the mean adipocyte Feret diameter was calculated for every patient.

### 4.5. RNA Isolation and cDNA Synthesis

The tissue samples for RNA isolation were homogenized using Precellys Lysing Kit CKMix Preps (Bertin Technologies, Montigny le Bretonneux, France) containing 1 ml TRI Reagent (Zymo Research, Irvine, CA, USA) and a TissueLyser LT (Qiagen, Hilden, Germany). The RNA was isolated using the Direct-zol™ RNA MiniPrep kit (Zymo Research, Irvine, CA, USA). cDNA was synthesized using SuperScript II reverse transcriptase (Life Technologies, Carlsbad, CA, USA) according to the supplier’s protocol.

### 4.6. Quantitative Real-Time Polymerase Chain Reaction

qPCR was performed using a LightCycler 2.0 and the LightCycler FastStart DNA MasterPLUS SYBR green I kit (both from Roche, Basel, Switzerland). Relative expression data were calculated using the 2^-ΔCT^-method and normalized to the geometric mean of the mRNA levels of 36B4 and HPRT as internal control genes. The following primers were acquired from Thermo Fischer Scientific (Ulm, Germany), with exception to the VEGFA primer, which was from Eurofins Scientific (Brussels, Belgium):

36B4 forw 5′-TGC ATC AGT ACC CCA TTC TAT CAT-3′;
36B4 rev 5′-AGG CAG ATG GAT CAG CCA AGA-3′;
ANGPT1 forw 5′-TGA GAC CCA GGT ACT AAA TCA AAC TTC TCG AC-3′;
ANGPT1 rev 5′-TGA AGA AGT TGC TTC TCT AGC TTG TAG GTG-3′;
ANGPT2 forw 5′- ACA GCA GAA TGC AGT ACA GAA CCA GAC G -3′;
ANGPT2 rev 5′-CAA GTC TCG TGG TCT GAT TTA ATA CTT GGG CT-3′;
FGF1 forw 5′-GAC CAG CAC ATT CAG CTG CAG CTC AGT G-3′;
FGF1 rev 5′-ACA AAC ATT CCT CAT TTG GTG TCT GTG AGC CG-3′;
FGF2 forw 5′-CGA CCC TCA CAT CAA GCT ACA ACT TCA AGC AG-3′;
FGF2 rev 5′-AGC CAG TAA TCT TCC ATC TTC CTT CAT AGC CA-3′;
HPRT forw 5′-GAG ATG GGA GGC CAT CAC ATT GTA GCC CTC-3′;
HPRT rev 5′-CTC CAC CAA TTA CTT TTA TGT CCC CTG TTG ACT GGT C-3′;
PDGFRa forw 5′-GAA TAA CAT CGG AGG AGA AGT TTC CCA GAG-3′;
PDGFRa rev 5′-CAT TTG GAA GGA TAG AGG GTA ATG AAA GCT GG-3′;
TEK forw 5′-CTA GAA GTA CAC CTG CCT CAT GCT CAG CC-3′;
TEK rev 5′-CAG TTC ACA AGC CTT CTC ACA CGT CCT TCC-3′;
VEGFA forw 5′-CTT GCC TTG CTG CTC TAC CT-3′;
VEGFA rev 5′-AGC TGC GCT GAT AGA CAT CC-3′;
VWF forw 5′-GAC CAA AGA GTC TCC ATG CCC TAT GCC T-3′;
VWF rev 5′-AGA TGC CCG TTC ACA CCA CTG TTC TCC A-3′.

To exclude artificial results due to outliers, expression values smaller or greater 3x, the standard deviation from the mean were excluded: VEGFA (*n* = 1), ANGPT1 (*n* = 1), ANGPT2 (*n* = 1), TEK (*n* = 1).

### 4.7. Statistical Analysis

Statistical analyses were performed using GraphPad Prism version 6.01. The significance level α for all analyses was set to 0.05. As most data did not follow a normal distribution, only the nonparametric Spearman correlation was performed. Trend lines were calculated as linear regressions.

## Figures and Tables

**Table 1:** Anthropometric parameters of the Ulm Childhood Adipose Tissue Collective. The data are given as mean ± SD, median, and range.

Parameter	*n*	Mean ± SD	Median	Range
Gender	45	36 male, 9 female		
Corrected age [y]	45	1.51 ± 2.18	0.24	−0.04–9.23
Weight [kg]	45	8.94 ± 6.30	5.62	2.07–25
Height [cm]	45	72.10 ± 24.83	61	42–131
BMI [kg·m^−2^]	45	15.16 ± 1.96	15.43	10.63–18.28
BMI-SDS	45	0.09 ± 1.08	0.06	−1.74–2.61
Adipocyte diameter [µm]	35	56.94 ± 10.63	56.66	39.73–88.89
Gender	35	28 male, 7 female		

**Table 2:** Results of the correlation analyses of the angiogenic factors in the Ulm Childhood comparing three age groups.

		Age		BMI-SDS		Adipocyte Diameter		VWF	
		*r_s_*	*p*	*r_s_*	*p*	*r_s_*	*p*	*r_s_*	*p*
VEGFA	all	0,262	0.086	−0.106	0.491	0.176	0.320	0.388	**0.009**
	<6 m	0.321	0.118	0.091	0.666	0.021	0.930	0.492	**0.013**
	≥6 m	–0.270	0.263	−0.282	0.241	0.218	0.454	0.260	0.283
FGF1	all	0.437	**0.003**	0.157	0.303	0.525	**0.001**	0.634	**<0.001**
	<6 m	0.687	**<0.001**	0.296	0.151	0.547	**0.012**	0.566	**0.003**
	≥6 m	−0.189	0.424	0.078	0.743	0.307	0.265	0.765	**<0.001**
FGF2	all	0.750	**<0.001**	0.017	0.913	0.429	**0.010**	0.418	**0.004**
	<6 m	0.665	**<0.001**	0.259	0.211	0.257	0.274	0.619	**0.001**
	≥6 m	−0.134	0.574	0.080	0.738	−0.050	0.863	0.535	**0.015**
ANGPT1	all	0.213	0.166	0.426	**0.004**	0.519	**0.002**	0.567	**<0.001**
	<6 m	0.353	0.090	0.494	**0.014**	0.226	0.352	0.483	**0.017**
	≥6 m	0.015	0.950	0.373	0.105	0.536	**0.042**	0.606	**0.005**
ANGPT2	all	−0.650	**<0.001**	0.148	0.337	−0.297	0.083	0.150	0.332
	<6 m	−0.038	0.861	0.067	0.756	0.003	0.990	−0.113	0.599
	≥6 m	−0.325	0.162	0.429	0.059	0.214	0.442	0.594	**0.006**
TEK	all	0.194	0.206	0.358	**0.017**	0.102	0.565	0.837	**<0.001**
	<6 m	0.572	**0.003**	0.480	**0.015**	0.281	0.230	0.742	**<0.001**
	≥6 m	−0.007	0.977	0.100	0.684	−0.064	0.832	0.881	**<0.001**

Total RNA was isolated, reverse transcribed, and subjected to qPCR analysis using specific primer pairs as indicated. Respective gene expression was calculated as described in Methods. Spearman correlation coefficient *r_s_* and *p* value, as well as number of subjects, are given for each analysis. Significant *p* values (*p* < 0.05) are indicated in bold.

**Figure A1:**
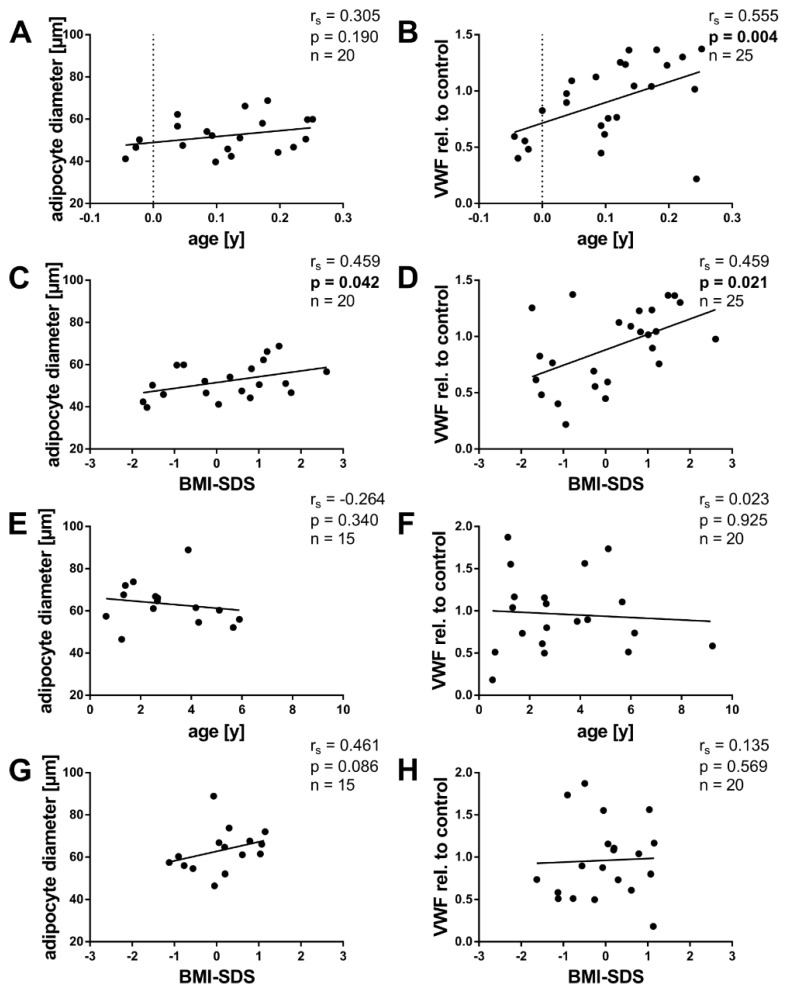
Association of adipocyte diameter and VWF expression with age and BMI-SDS in the WAT of infants <6 and children ≥6 months. Adipocyte size was determined in histologic sections as specified in Methods. Total RNA was isolated, reverse transcribed, and subjected to qPCR analysis using a specific primer pair as indicated. VWF gene expression was calculated as described in Methods and plotted against age. (**A**) In contrast to the analysis with all children, adipocyte size did not correlate positively with age in the infants <6 months. (**C**) Adipocyte size did, in turn, correlated positively with BMI-SDS. (**B**,**D**) VWF correlated positively with age and BMI-SDS in the infants <6 months. (**E**–**F**) There were no significant correlations of adipocyte diameter and VWF with age or BMI-SDS in the children ≥6 months. Spearman correlation coefficient *r_s_* and *p* value, as well as number of subjects, are given in each scatter plot. Significant *p* values (*p* < 0.05) are indicated in bold. Trend lines were calculated as linear regressions.

## Abbreviations

36B4	Acidic ribosomal phosphoprotein P0
ANGPT1	Angiopoietin-1
ANGPT2	Angiopoietin-2
BMI	Body mass index
FGFs	Fibroblast growth factors
H&E	Haematoxylin and eosin
HPRT	Hypoxanthine-guanine phosphoribosyltransferase
mRNA	Messenger ribonucleic acid
PDGFRa	Platelet-derived growth factor receptor alpha
qPCR	Quantitative polymerase chain reaction
RNA	Ribonucleic acid
SD	Standard deviation
SDS	Standard deviation score
SVCs	Stromal vascular cells
TEK (TIE-2)	Tunica interna endothelial cell kinase, an angiopoietin receptor
VEGFA	Vascular endothelial growth factor A
VWF	Von Willebrand factor
WAT	White adipose tissue
WHO	World Health Organization
